# Case Report: Acute Necrotizing Encephalopathy Following COVID-19 Vaccine

**DOI:** 10.3389/fneur.2022.872734

**Published:** 2022-04-29

**Authors:** Mohamed Reda Bensaidane, Vincent Picher-Martel, François Émond, Gaston De Serres, Nicolas Dupré, Philippe Beauchemin

**Affiliations:** ^1^Department of Medicine, Faculty of Medicine, Centre Hospitalier Universitaire de Québec—Université Laval, Quebec, QC, Canada; ^2^Department of Psychiatry and Neuroscience, Faculty of Medicine, CERVO Brain Research Centre, Quebec, QC, Canada; ^3^Institut National de Santé Publique du Québec, Quebec, QC, Canada

**Keywords:** COVID, acute necrotizing encephalopathy, vaccine, Neuroimmunology, ANE, akinetic mutism, encephalopathy

## Abstract

**Objectives:**

Acute necrotizing encephalopathy (ANE) is a rare neurological disorder arising from a para- or post-infectious “cytokine storm. ”It has recently been reported in association with coronavirus disease 2019 (COVID-19) infection.

**Methods:**

A 56-year-old male with a diagnosis of ANE 48 h following the first dose of ChAdOx1 nCoV-19 vaccination was investigated. Cytokine analyses on serum and cerebrospinal fluid (CSF) were performed. The patient was treated with high-dose corticosteroids and followed clinically and radiologically.

**Results:**

Favorable clinical and radiological outcomes were noted. There was an upregulation in serum levels of CXCL5, CXCL1, Il-8, IL-15, CCL2, TGF-B, and EGF, and up-regulation in CSF levels of CXCL5, IL-2, IL-3, and IL-8.

**Discussion:**

As COVID-19 infection has been previously reported as a possible rare cause of ANE, we speculate on an aberrant immune response mechanism that was brought about by the vaccine. To increase our understanding of the pathogenesis of ANE in the context of COVID-19 vaccination and to better define its clinical features and outcomes, clinicians and scientists should continue reporting convincing cases of such entities.

## Introduction

We case report of a 56-year-old male who was found to have altered mental status upon awakening. The patient had no specific complaints the day prior aside from mild fatigue. He had received his first dose of the ChAdOx1 nCoV-19 vaccine 2 days prior. He had no viral prodrome in the weeks preceding his vaccination. His past medical history was significant for hypertension and a self-limited viral myocarditis 2 years before.

Upon his arrival to the emergency department, the patient's vital signs showed blood pressure at 160/100 mmHg and a rectal temperature of 38.3°C. His neurological examination was compatible with a state of akinetic mutism. Initial laboratories revealed white blood cell count (WBC) at 11.07 × 10^9^/L. The toxicology screen workup was negative. The RT-PCR COVID-19 test was negative. Computed tomography angiography (CTA) with venous phase of the brain and neck was normal. Electroencephalogram (EEG) showed mild diffuse slowing but with no epileptiform discharges.

**Figure 1:**
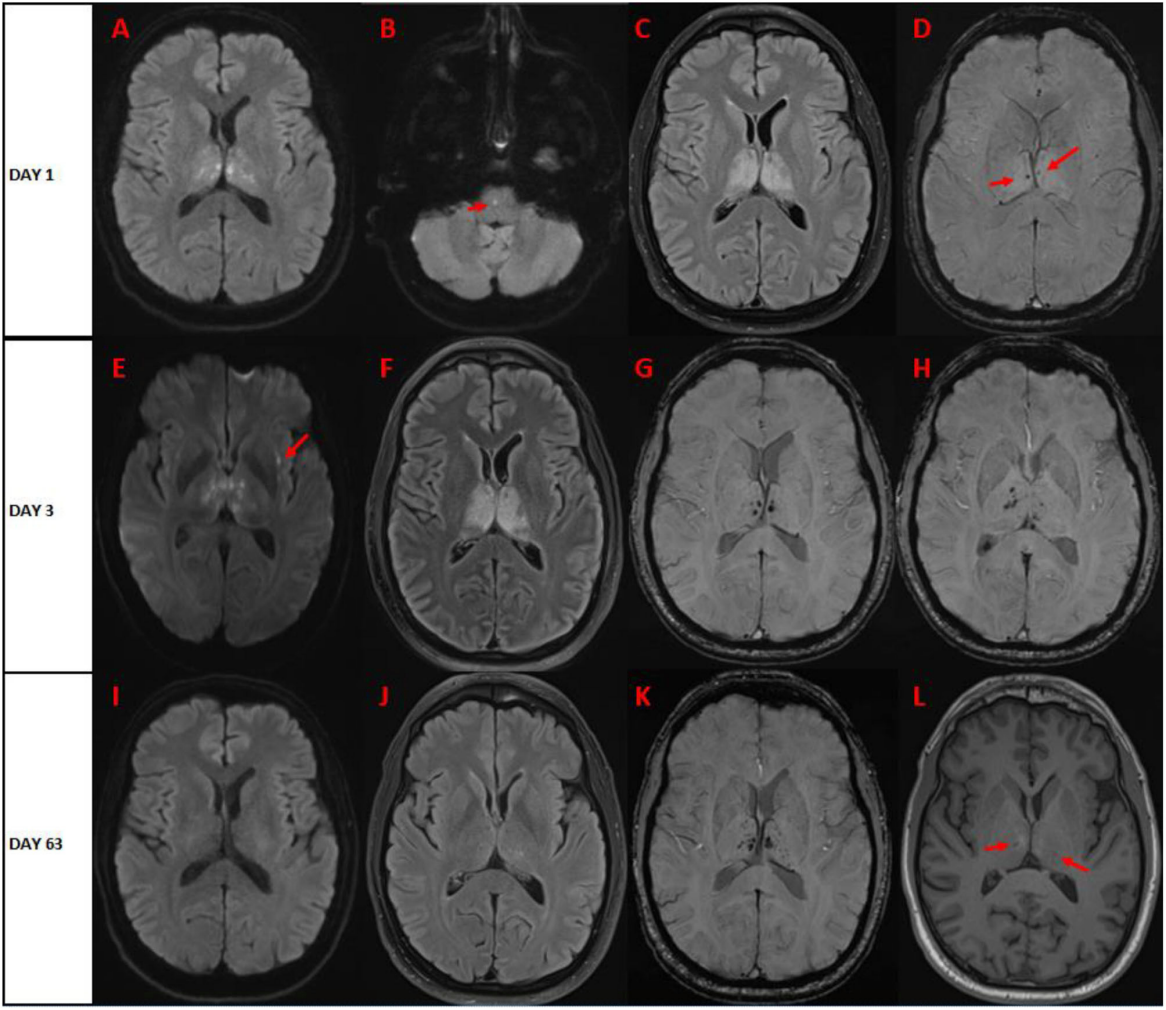
Brain magnetic resonance on **(A–D)** *day* 1 **(E–H)** day 3, and **(I-L)** day 63 after symptom onset. **(A)** Diffusion-weighted imaging (DWI) shows bilateral symmetric punctiform restrictive lesions in bilateral thalami. **(B)** DWI shows single punctiform restrictive pontine lesion. **(C)** FLAIR imaging shows bilateral hyperintense lesions of the thalami, with no significant mass effect. **(D)** Susceptibility-weighted imaging (SWI) shows two punctiform hypointense lesions in bilateral thalami. On day 3, **(E)** DWI shows increase of the restrictive lesions of bilateral thalami, and new peri-insular restrictive lesions (arrow). **(F)** FLAIR imaging shows relative stability of bilateral hyperintensities involving thalami. **(G,H)** SWI shows increase hypointense microhemorrhagic lesions of bilateral thalami. On day 63, **(I)** DWI sequence shows resolution of restrictive lesions. **(J)** FLAIR imaging sequence reveals near resolution of thalamic hyperintensities. **(K)** SWI indicates persistence of hypointense microhemorrhagic bilthalamic lesions. **(L)** T1-weighted imaging shows subtle hypointense lesions in bilateral thalami (arrows).

Magnetic resonance imaging (MRI) of the brain performed on day 1 (Figures 1A-D) showed hyperintensities involving bilateral thalami, with some diffusion restriction and microhemorrhagic components. Repeat brain MRI on day 3 showed relative stability of the FLAIR lesions, with increase in the hemorrhagic component within the thalami (Figures 1E-H). There was no enhancement post-gadolinium injection.

Cerebral spinal fluid (CSF) analysis on day 1 showed no WBC, and slightly increased proteins at 0.84 g/L. Multiplex PCR for infectious causes (HSV-1/2, HHV-6, HpeV, VZV, enterovirus, CMV, listeria, and cryptococcus) was negative. Repeat lumbar puncture on day 4 showed similar results. Oligoclonal bands were negative. In addition, rheumatological workup was non-contributory. Anti-aquaporin-4 and anti-myelin-oligodendrocyte antibodies were negative.

High-dose pulse intravenous corticosteroids (methylprednisolone 1g IV daily for 7 days) were started on day 4 and were followed by prednisone taper. Progressive clinical improvement was noted in the following days, with improvement in motor, visuospatial, language, and social skills. He was discharged to the rehabilitation after 4 weeks. Prednisone was tapered off over a period of 5 weeks. On follow-up 6 weeks after discharge, the patient made a near-complete recovery, with no apparent sequelae. Favorable radiological evolution was noted (Figures 1I-L).

In total, the patient had three negative RT-PCR COVID-19 tests, as well as two negative COVID-19 serologies against spike and nucleocapsid proteins (performed 2 and 6 weeks after admission). Ran-binding protein 2 (RANBP2) gene sequencing with copy variant analysis was performed, as pathogenic mutations have been described to cause familial and recurrent forms of acute necrotizing encephalopathy (1). The results revealed a heterozygous c.8293-10C > T variant in intron 23. *In silico* splicing algorithms did not predict the adverse effect of this variant on splicing.

**Figure 2:**
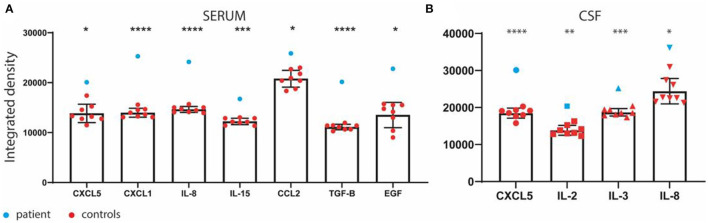
**(A)** Serum and **(B)** cerebrospinal fluid (CSF) cytokine profile in the patient and eight healthy age-matched controls from a local biobank. *p ≤ 0.05; **p ≤ 0.01; *** *p* ≤ 0.001; ****p ≤ 0.0001.

We analyzed the cytokine profile in blood and CSF drawn on day 4 of admission, thus prior to pulse corticosteroid therapy. We used an unbiased approach by cytokines array (Raybiotech) to study the protein levels of 42 cytokines. We compared these levels with eight healthy aged-matched controls from a local anonymized biobank. We observed upregulation in serum levels of CXCL5 (*p* = 0.0173), CXCL1 (*p* < 0.0001), Il-8 (*p* < 0.0001), IL-15 (*p* = 0.0004), CCL2 (*p* = 0.0288), TGF-B (*p* < 0.0001), and EGF (*p* = 0.0117) (Figure 2A). There was upregulation in CSF levels of CXCL5 (*p* < 0.0001), IL-2 (*p* = 0.0026), IL-3 (*p* = 0.0004), and IL-8 (*p* = 0.0145) (Figure 2B). We did not observe any significant changes in TNF-α, IFN-γ, IL-4-5-6-7-10, IL-1β, CCL1-5-17-22, and G-CSF, which were previously reported to be upregulated under similar conditions (2, 3).

## Discussion

Given the clinical and radiological evolution of the case, the patient's final diagnosis was established as being acute necrotizing encephalopathy (ANE). ANE is an extremely rare condition affecting mostly children and results from a para- or post-infectious “cytokine storm” that leads to breakdown of the blood-brain barrier and subsequent central nervous system (CNS) insult (1). The condition is frequently associated with signs of systemic inflammatory response syndrome (SIRS) and may evolve into shock, multiple organ failure (MOF), or disseminated intravascular coagulation (DIC). Proposed diagnostic criteria for ANE (4) are shown in Table 1. Our patient meets all the criteria except for elevation of serum aminotransferases. Such finding was frequently observed in case series of ANE and has, hence, been incorporated into the criteria as a marker of systemic symptoms that can arise from the immune response that leads to ANE. It is, however, not a universal feature, and we do not consider it to preclude the diagnosis. Furthermore, we note that our patient's case was not preceded by a viral febrile illness.

**Table 1:** Diagnostic criteria for acute necrotizing encephalopathy (ANE) contrasted to our patient's case.

**Diagnostic criteria**	**Our patient**
(1) Acute encephalopathy preceded by viral febrile disease; rapid deterioration in the level of consciousness or convulsions.	Fulfilled
(2) Increased CSF protein without pleocytosis.	Fulfilled
(3) CT or MRI evidence for symmetric, multifocal brain lesions. Involvement of the bilateral thalami. Lesions also common in the cerebral periventricular white matter, internal capsule, putamen, upper brainstem tegmentum and cerebellar medulla. No involvement of other CNS regions.	Fulfilled
(4) Elevation of serum aminotransferases of variable degrees. No increase in blood ammonia.	Unfulfilled
(5) Exclusion of other resembling diseases.	Fulfilled

Radiologically, the hallmark feature of ANE is bilateral thalamic involvement, which is found in all cases of ANE (1, 4). Other multifocal brain lesions are usually present as shown in the diagnostic criteria. Such lesions have been described to have dynamic changes during the clinical course from edema to petechial hemorrhage and then to necrosis (5). Significant regression of these brain lesions has been reported in survivors of ANE. Our case highlights these imaging findings and their evolution (Figure 1). Furthermore, differential diagnosis of acute bilateral thalamic lesions should be thoroughly assessed by clinicians. These include ischemic strokes (“top of the basilar” occlusion or artery of Percheron infarcts), deep venous occlusions, vasculitis, hypoxic-ischemic encephalopathy, acute disseminated encephalomyelitis (ADEM), Fabry disease, Wernicke encephalopathy, osmotic demyelination, viral encephalitis (Japanese encephalitis, West Nile virus, dengue virus, and others), neoplasms (lymphomas and astrocytomas), and Creutzfeldt-Jakob disease. Cases of autoimmune and paraneoplastic encephalitis harboring bilateral thalamic involvement are also increasingly being reported (6, 7). In children, differential diagnosis also includes Leigh Syndrome and inborn errors of metabolism.

ANE is distinct from inflammatory and demyelinating conditions that can occur in post-infectious or post-vaccinal states. Neuropathological cases of ANE demonstrated the absence of demyelination and important parenchymal abnormalities (perivascular hemorrhage and necrosis and vasogenic edema), with no significant inflammatory cell infiltration (4, 8, 9).

Previously reported cytokine profiles in influenza-associated ANE were mostly characterized by IL-6 and TNF-α increase (2), although this was not invariable (3). The immune response to the SARS-CoV-2 vaccine is driven by neutralizing antibodies and antigen-specific T cells. It was demonstrated that ChAdOx1 the nCoV-19 vaccine induces a Th1-biased response mostly characterized by production of interferon-y (IFN-y) and IL-2, and a small increase in IL-10 (10).

Some studies in ADEM suggested a CSF increase in Th1-related cytokines (TNF-α, IL-657 2, IFN-γ), Th2-related (IL-4, IL-5), macrophages-related (IL-1β, CCL4, IL-6, IL-8, G-CSF) and in chemokines (CXCL1-7-10 and CCL1-3-5-17-22) (11). Interestingly, the most consistent changes observed in our patient's serum and CSF samples were increase in CXCL5 and IL-8, which are both members of the ELR(+) CXC chemokine family. It was recently suggested that infection of human-induced pluripotent stem cell (iPSC) neural cultures by SARS-CoV-2 may induce IL-8 production (12). Although our patient's CSF and serum cytokine profile did not correspond directly to any previously described cytokine profile in ANE, it may suggest aberrant activation of mononuclear cells driving a CNS inflammatory process. The main limitations of our cytokine analyses include the fact that they were performed at only a single time point, and that they were performed 4 days after admission.

Several cases of ANE have been reported in adult patients with active COVID-19 infection (13, 14). Cases of CNS demyelination syndromes have been described in patients infected with SARS-CoV-2 (13). These include the clinically aggressive variant of ADEM, namely, acute hemorrhagic encephalomyelitis (AHEM). The interest in our case lies in the fact that the patient had no clinical or laboratory evidence of SARS-CoV-2 infection. The only potential precipitating factor was the administration of the ChAdOx1 nCoV-19 vaccine 2 days prior. Interestingly, there is a recently published case of a 75-year-old woman who developed ANE 2 days after receiving the ChAdOx1 nCoV-19 vaccine (15). Unlike our patient, however, the patient's outcome was unfavorable, as she died 1 month after disease onset despite corticosteroid treatment. Also, cases of AHEM within the first few days of the first shot of the ChAdOx1 nCoV-19 vaccine have been described (16). Although ANE and AHEM share clinical similarities in their aggressiveness and severity, key radiological, neuropathological, and laboratory differences allow for the distinction of the two entities (4, 17). Whether these rare conditions share a common pathophysiological basis remains a matter of debate, as some have considered them to occur as part of a continuum (13). Finally, cases of autoimmune encephalitis following ChAdOx1 nCoV-19 have been reported (18, 19). Such cases of altered sensorium following this vaccine are summarized in Table 2.

**Table 2:** Cases presenting with altered level of consciousness following ChAdOx1 nCoV-19 vaccine.

**Author/ country**	**Age/ gender**	**Relevant co-morbidity**	**Time from vaccination to symptom onset**	**Neurological presentation**	**Initial MRI findings**	**CSF findings**	**Diagnosis**	**Proposed mechanism**	**Management**	**Outcomes**
Our case/Canada	56 y/M	Viral myocarditis 2 years prior	2 days after 1st dose	Fever and altered level of consciousness (akinetic mutism)	T2/FLAIR hyperintensities in thalami. Scattered punctate foci of diffusion restriction and petechial haemorrhage	Proteins at 0.84 g/L Normal cell count	ANE	Aberrant immune response	IVMP 1g/d × 7 days, followed by a 5-week prednisone taper	Full recovery after 6 weeks of rehabilitation
Siriratnam et al. (15)/Australia	75 y/F	Eosinophilic granulomatosis with polyangiitis; ceased all immunotherapy 12 months prior to illness Monoclonal gammopathy of unknown significance	2 days after 1st dose	Altered level of consciousness, dysarthria, followed by seizures	T2 hyperintensities in thalami and medial temporal lobes. Scattered punctate foci of diffusion restriction and petechial haemorrhage	Proteins at 2.98 g/L Normal cell count	ANE	None	IVIg 2 g/kg × days and intravenous methylprednisolone 1 g/d × 4 days, followed by prednisolone 1 mg/kg × 3 weeks	Death 1 month after disease onset
Permezel et al. (26)/Australia	63 y/M	Insulin-dependant type II diabetes mellitus Ischemic heart disease Atrial fibrillation	12 days after 1st dose	Vertigo, abdominal pain, fatigue, followed by decrease of consciousness after 4 days.	Bilateral white matter T2 hyperintensities in periventricular and juxtacortical areas	Initially no pleocytosis, protein 0.69 g/L. Subsequent CSF: 8 cells (mononuclear).	ADEM (proved by post-mortem neuropathology)	None	IVMP 1 g/d × days, followed by PLEX.	Death 20 days after admission
Ancau et al. (16)/Germany (case 1)	61 y/M	Hypothyroidism and polymyalgia rheumatica	2 days after 1st dose	Fever, headache and apathy followed by loss of consciousness and seizures	Bilateral confluent cortical and subcortical FLAIR lesions with hemorrhagic involvement of basal ganglia, specifically thalami	Normal cell count	AHEM	Molecular mimicry or re-infectious etiology	IVMP 1g/d × 5 days, followed by seven PLEX sessions with concomitant methylprednisolone 250 mg *via* nasogastric tube	Vegetative state after 14 weeks of rehabilitation
Ancau et al. (16)/Germany (case 3)	55 y/F	Unspecified	9 days after 1st dose	Nausea, dizziness and meningismus, progressing rapidly to spastic tetraparesis and coma	Multiple FLAIR hyperintensities with hemorrhagic lesions in the right parietal and temporal lobes, bilateral fronto-temporal regions, and right occipital lobe and left fronto-basal region	Lymphocytic pleocytosis (10 cells)	AE		IVMP 1g/d × 5 days followed by methylprednisolone 100 mg *via* nasogastric tube taper. Repeat treatment following deterioration of edema and increase of hemorrhagic lesions	Death
Chakrabarti et al. (27)/India	60 y/F	None	1 day after 2nd dose	Confusion, forgetfulness, hallucinations progressing over 5 days, followed by rigidity	FLAIR hyperintensities in bilateral caudate heads which showed diffusion restriction. Patchy diffusion restriction in left posterior parietal and occipital gyri. Deterioration of abnormalities on repeat imaging	Normal	Post-vaccinal prion-like neurodegeneration	Toxicity of S protein or toxicity of anti-S protein antibodies	Dexamethasone, antiepileptics, broad-spectrum antibiotics	Death 1 month after disease onset
Kwon et al. (19)/Korea	57 y/F	Hypertension	5 days after 2nd dose	Generalized seizure. Had fever and headache in the preceding days	Left insular and mesial temporal cortices restriction diffusion. 2 months after, encephalomalacia change in the affected temporal lobe	Initially normal 22 cells (91% lymphocytes) on repeat CSF 1-month after, with proteins at 88.3 mg/dl	AE	Central nervous system autoimmunity	Initially acyclovir and anticonvulsants. Methylprednisolone, IVIg and Rituximab after 1 month	Significant memory deficits
Maramattom et al. (28)/India (Case 1)	64 y/M	Unknown	10 days after 1st dose	Headache, fever, and drowsiness	FLAIR hyperintensities in mesial temporal lobe and middle cerebellar peduncles	Lymphocytic pleocytosis (value not provided)	AE (LE)	None	IVMP 1g/day × 5days and PLEX. Rituximab after 8 weeks	Discharged with no deficits; mRS 1
Maramattom et al. (28)/India (Case 2)	65 y/M	Unknown	10 days after 2nd dose	Behavioral changes. Developed jerky movement over the next 3 weeks	Unknown	Mild pleocytosis (10 cells)	OMAS	None	IVMP 1 g/d and IVIg for 5 days	mRS 1
Zuhorn et al. (18)/Germany (Case 1)	21 y/F	Obesity	5 days after 1st dose	Headache, attention and concentration difficulties. Seizures and stupor afterward	Normal	Lymphocytic pleocytosis (46 cells)	AE	Aberrant immune response	Dexamethasone 10 mg/d	Mild cognitive slowing at discharge with no functional impairment
Zuhorn et al. (18)/Germany (Case 2)	63 y/F	Deep vein thrombosis 2 days after vaccination	6 days after vaccination. Unspecified if 1st or 2nd dose	Gait deterioration with vigilance impairement and twitching, followed by opsoclonus-myoclonus syndrome	Normal	Lymphocytic pleocytosis (115 cells)	OMS		IVMP 1 g/d × 5 days.	Immediate improvement following treatment. Low-grade tremor as the only residual neurological deficit
Zuhorn et al. (18)/Germany (Case 3)	63 y/M	Unspecified	8 days after vaccination Unspecified if 1st or 2nd dose	Fever, headache and reduced alertness	Normal	Lymphocytic pleocytosis (7 cells)	AE		No treatment	Spontaneous improvement

*AE, autoimmune encephalitis; ADEM, acute demyelinating encephalomyelitis; AHEM, acute hemorrhagic encephalomyelitis; ANE, acute necrotizing encephalopathy; CSF, cerebrospinal fluid; IVMP, intravenous methylprednisolone; LE, limbic encephalitis; MRI, magnetic resonance imaging; mRS, modified Rankin Scale; OMS, opsoclonus-myoclonus syndrome; OMAS, opsoclonus-myoclonus ataxia syndrome; PLEX, plasmapheresis*.

Although post-vaccinal CNS demyelination syndromes tend to occur at a mean of 14-days following vaccination (20), post-vaccinal timing of ANE is unclear because of its rarity. A case of ANE 6 days after diphtheria, tetanus toxoid, and whole-cell pertussis (DTPw) vaccination was reported in a previously healthy 6-month-old boy (21). Since our case shares similar timing to the previously reported case of ANE (15), and the fact that ANE is not considered a demyelinating condition, we consider the timing of the vaccine to be plausible. Furthermore, the ChAdOx1 nCoV-19 vaccine uses an adenovirus vector (22). Rapid induction of the innate immune system has been described with adenovirus viral vectors and could be IL-8-mediated (23). Hence, a hypothetical mechanism for ANE in our patient could include vector-induced aberrant immune response. However, since ANE has been reported as a rare manifestation of COVID-19, we can also speculate on a rapidly evolving aberrant immune response mechanism or even a molecular mimicry mechanism that could have been induced by the vaccine-contained SARS-CoV-2 spike protein epitope. Finally, the ChAdOx1 nCoV-19 vaccine does not contain adjuvants, ruling out such an immunopathogenic mechanism (22).

Our patient had negative COVID-19 serologies against spike and nucleocapsid proteins despite vaccination. We attribute the negative serologies to the administration of pulse corticosteroid therapy that was begun 5 days after vaccination, followed by high-dose prednisone taper. Immunogenicity data of the ChAdOx1 nCoV-19 vaccine show that antibodies against SARS-CoV-2 spike proteins significantly increase after 14 days and peak after 28 days, with no significant difference between day 0 and day 7 (24). Hence, with these elements, we suggest that our patient's treatment blunted the immune response to the vaccine and explains the negative serologies. Rapid treatment may have influenced the positive outcome in our patient's situation, as has been retrospectively shown in children with ANE (25).

Of note, RANBP2 mutations were found to predisposed to recurrent episodes of ANE in children (1). The mutation found in our case (c.8293-10C > T**)** has never been described in the literature to our knowledge. As an intronic point mutation with no expected splicing consequences, it is most likely to be unrelated to our patient's condition and is expected to be benign.

In summary, we report a case of ANE occurring after the first dose of the ChAdOx1 nCoV-19 vaccine. The patient was promptly treated with corticosteroids and had a very favorable outcome. To increase our understanding of the pathogenesis of ANE in the context of COVID-19 vaccination and to better define its clinical features and outcomes, clinicians and scientists should continue reporting convincing cases of such entities.

## Data Availability Statement

The datasets presented in this article are not readily available because of ethical and privacy restrictions. Requests to access the datasets should be directed to the corresponding author/s.

## Ethics Statement

The studies involving human participants were reviewed and approved by CER du CHU de Québec-Université Laval. The patients/participants provided their written informed consent to participate in this study. Written informed consent was obtained from the individual(s) for the publication of any potentially identifiable images or data included in this article.

## Author Contributions

MB has done the required academic literature review for the conception of the article and drafted the article. VP-M has performed laboratory cytokine testing as well as critical revision of the article. FÉ, GS, and ND have performed critical revision of the article. PB has done a critical revision of the article and performed the final approval of the version to be published. All authors contributed to the article and approved the submitted version.

## Conflict of Interest

The authors declare that the research was conducted in the absence of any commercial or financial relationships that could be construed as a potential conflict of interest.

## Publisher's Note

All claims expressed in this article are solely those of the authors and do not necessarily represent those of their affiliated organizations, or those of the publisher, the editors and the reviewers. Any product that may be evaluated in this article, or claim that may be made by its manufacturer, is not guaranteed or endorsed by the publisher.
